# Supplementary material for: Optimization of Constitutive Promoters Using a Promoter-Trapping Vector in Burkholderia pyrrocinia JK-SH007
Source: Int J Mol Sci. 2023 May 29;24(11):9419. doi: 10.3390/ijms24119419 (PMC10253294; doi:10.3390/ijms24119419)
Supplement: Supplementary file 1 [file ijms-24-09419-s001.zip › ijms-2364827-supplementary.pdf]

Optimization of Constitutive Promoters Using a Promoter-Trapping Vector in *Burkholderia pyrrocinia* JK-SH007

Xue-Lian Wu [1,2,†], Xiao-Wei Liu [1,2,†], Yang Wang [3], Meng-Yun Guo [4] and Jian-Ren Ye [1,2,*]

1 Co-Innovation Center for Sustainable Forestry in Southern China, College of Forestry, Nanjing Forestry University, Nanjing 210037, China; xuelianwuletter@163.com (X.-L.W.); njfulxw@163.com (X.-W.L.)

2 Jiangsu Key Laboratory for Prevention and Management of Invasive Species, Nanjing Forestry University, Nanjing 210037, China

3 Institute of Forest Pest Control, Jiangxi Academy of Forestry, Nanchang 330032, China; wy21sj@163.com

4 Key Laboratory for Bio-Resources and Eco-Environment of Ministry of Education, College of Life Science, Sichuan University, Chengdu 610065, China; mengyun_guo@163.com

* Correspondence: jrye@njfu.edu.cn

† These authors contributed equally to this work.

SUPPLEMENTARY MATERIAL

Supplementary Tables

Table S1 Primers used in this study.
Table S2 Promoter sequences selected in this study.

Supplementary Figures

Figure S1 Trap vector with two reporter genes.
Figure S2 Growth curves of *B. pyrrocinia* JK-SH007.
Figure S3 Growth curves of *E. coli* S17-1.
Figure S4 Assay of constitutive promoters from *E. coli* S17-1 using firefly luciferase.
Figure S5 Promoter characterization via qPCR analysis in *E. coli* S17-1.
Figure S6 Growth curves of *B. multivorans* WS-FJ9.
Figure S7 Assay of constitutive promoters from *B. multivorans* WS-FJ9 using firefly luciferase.
Figure S8 Promoter characterization via qPCR analysis in *B. multivorans* WS-FJ9.

Table S1. Primers used in this study.

Primers	Primer sequences (5'-3')	Source
P4-F	*GGCCTTTCGTTTTATAAGCTT*TTCCTCCTCTAGGGAGGTG	pBBR1-GM^{r}-P4-Luc-TP^{r}
P4-R	*TGGTAAATTACTCGAGGCGCC*AACTGCGGTAGCTGCAATGA	
P9-F	*GGCCTTTCGTTTTATAAGCTT*GGCTCACCGGCCGGGC	pBBR1-GM^{r}-P9-Luc-TP^{r}
P9-R	*TGGTAAATTACTCGAGGCGCC*CCACAGTACCGCGAAGGAT	
P10-F	GGCCTTTCGTTTTATAAGCTTCAACCGGCTGCGCATTCC	pBBR1-GM^{r}-P10-Luc-TP^{r}
P10-R	*TGGTAAATTACTCGAGGCGCC*GGTGTCCTCGGATTTGTGC	
P14-F	*GGCCTTTCGTTTTATAAGCTT*TTTCCGCACGCCGGCGCCG	pBBR1-GM^{r}-P14-Luc-TP^{r}
P14-R	*TGGTAAATTACTCGAGGCGCC*CGGGCACGCTCCTGTTGG	
P15-F	*GGCCTTTCGTTTTATAAGCTT*GCGGACGGTTCCAAAAACG	pBBR1-GM^{r}-P15-Luc-TP^{r}
P15-R	*TGGTAAATTACTCGAGGCGCC*ACAACCACCTTCGAATCCTTT	
P17-F	*GGCCTTTCGTTTTATAAGCTT*CCCGCAGCAGCGAACCC	pBBR1-GM^{r}-P17-Luc-TP^{r}
P17-R	*TGGTAAATTACTCGAGGCGCC*GTTGTTCTCCTTCAGGTTCAG	
P18-F	*GGCCTTTCGTTTTATAAGCTT*CGACTTGATCGAACCCGTG	pBBR1-GM^{r}-P18-Luc-TP^{r}
P18-R	*TGGTAAATTACTCGAGGCGCC*GTCCGTTTCTTGCTGATTCTG	
P19-F	*GGCCTTTCGTTTTATAAGCTT*CCCGCCCGCAAGCGCC	pBBR1-GM^{r}-P19-Luc-TP^{r}
P19-R	*TGGTAAATTACTCGAGGCGCC*AAGATTCCGTTTTTGCATCGTT	
PT1-F	*TCGACCTCGAGGGGGGGCCC*GGTACCCAGCTTTTGTTCCCTTTA	pBBR1-GM^{r}-PRPL larger fragment [1]
PT1-R	CAGCCCGGGGGATCCACTAGT*TCTAGAGCGGCCGCCA*	
PRPL-F	GCGCGTAATACGACTCACTATAG	*PrPl* smaller fragment [2]
PRPL-R	*TTGGCATCTTCCATG*TATGAGTCTCCAGTTTGTTTCAGTT	
Luc-TP^{r}-F	*CTGAAACAAACTGGAGACTCATA*CATGGAAGATGCCAAAAACATTAAG	Luc-TP^{r} smaller fragment [2]
Luc-TP^{r}-R	*TAAAGGGAACAAAAGCTGGGTACC*GGGCCCCCCCTCGAG	
PTC1-F	GCGCGTAATACGACTCACTATAG	pBBR1-GM^{r}-PRPL-Luc-TP^{r} colony PCR check
PTC1-R	GGCTCGTATGTTGTGTGGAA	
PT2-F	GGCGCCTCGAGTAATTTA	pBBR1-GM^{r} -Luc-TP^{r} larger fragment
PT2-R	AAGCTTATAAAACGAAAGGCC	
PTC2-F	GCGCGTAATACGACTCACT	pBBR1-GM^{r}-Promoter-Luc-TP^{r} colony PCR check
PTC2-R	ATGTTGCATCACCTTCACC	
PT3-F	GAATTCTTTGCTTGTCGCC	pBBR1-GM^{r} -GFP-TP^{r} larger fragment *gfp* fragment
PT3-R	TATGAGTCTCCAGTTTGTTTCAGTT	
gfp-F	*AACAAACTGGAGACTCATA*ATGAGTAAAGGAGAAGAACT	
gfp-R	*TTATGGCGACAAGCAAAGAATTC*TTATTTGTATAGTTCATCCAT	
rfp-F	*TGAAACAAACTGGAGACTCATA*ATATAGCATGCGGGTCTTCCAA	*rfp* fragment
rfp-R	*ATTATGGCGACAAGCAAAGAATTC*TTAAAGGAACAGATGGTGGC	
qRT-*luc*-F	GCTCAGCAAGGAGGTAGGTG	qPCR for *luc* gene
qRT-*luc*-R	CCAGTGTCTTACCGGTGTCC	
qRT-*pyr*G-F	AGTCACCCTCCTCAAACTCG	qPCR for *pyrG* gene [3]
qRT-*pyr*G-R	TCGTGAAGTTGTTGGCCTTG	
qRT-16SrRNA-F	GATGACCAGCCACACTGGAA	qPCR for *E.coli* 16SrRNA gene
qRT-16SrRNA-R	GGAGTTAGCCGGTGCTTCTT	
qRT-16SrRNA-F	CCTACCAAGGCGACGATCAG	qPCR for *B.multivorans* 16SrRNA gene
qRT-16SrRNA-R	CAAAATTCCCCACTGCTGCC	

The homology arms are in italic.

Table S2. Promoter sequences selected in this study.

Promoter	Promoter sequence
P4	CATTTCCTCCTCTAGGGAGGTGGGCAGGGTCTCTTTGGCGAATTGATCCATGTATCCGTATCGTTTCGGAGCG<br>AAGACGGCGAACGCCTTTCGCGTGGGCGCTGCATGCGCAACGCAACGCGAGATTCTAACATGCGCCCATC<br>AGCCGCAAATTCGCCTCCCCCTCTATATATAAATAGCGAAAATCGCGGCGGCGGCGGACCGGCCGGAACCG<br>CACGCGACTCGACGCCACCGTTGATCGCGCCCGGTTTACCGGCGATCCCACCCGGTTTGTTTCACGCGCAC<br>GAGTAAACAGTTGCCCGCACCCGCGCAATTTCGCCGGCGCACTCTTGACAATCTCTAAAAGTTGCGGCAGG<br>CCGGTGTTGCGCATGCACCACAAAGTTGAGGGGATCTTAGGGTGGGGAGGATTTTTTTCGTAGGAAGGGA<br>ACGATATGGCAAAATGCCAACGCACTGAAAGTTAGACGCTGCTCGAAGTCTACACAGAGCGGTGCCGCGT<br>TTTTTGTGCCGCACCAATGTTATACTTCGAGCAATGTATGACTTGTCACCGTCAACAGGCTCGCAGTAAACC<br>TGCGGTAATCTCAATTTCGAGAGGAGAAATATGAATAAACTTTCAAAGCTCGCGTTCATTGCAGCTACCGCA<br>GTTATG
P9	CACGGCTCACCGGCCGGGCCGGGGCTGACGCGCGCCCCGGCAGTCCCGATCTGGCATACGGCCCGCGATC<br>GCGTCGATCGCACCTCTGGCACGAGGCGGCGGCAGCCGCCGAGGACGGGCGTCCGCGGCACAGGATCGG<br>GAAGTTCCCAGTGTGGGGCCCGATCGCGTGCAAGTCTGTCCCGAAAGTTTCGTGAAAGTGTGCCGATTTGT<br>GCGCGATTGTCGGAGTGTGTCGTTCCGGCCGCGCCGTCAAAACGCTGAAACATCGGGTCGGACCGCATCCC<br>GGCAGTGCCGGATTGCGTCGATCGGCCCGGTTCCGGCGGCGGCCCGAGCGCGACAGATCGACGCGCCCCC<br>GGCCATGTCATCCCATGTCTCGAACGTCGCCGATGCTTGCGTCGACACCGCAAGCGCTGATGCCACGTGCG<br>TTTGCGCGCGCCGCCGAAATGTCCGCGGATCGTCAGACGTCGTTTCATCTGAAACCGCAATGGACTCAGTAT<br>CGATTTATTGCGGATTATCGCTTGCGTTTCACACCAGTCTATCTAGACTATTCAAGTCCAAACGGTCAAATTC<br>GTCCTTTTTTCAGGAGACGATCCAATGCGCCTCACCATTCGAATTAATGGCAGCGAATCGGCAACCCGGCA<br>ATCCTTCGCGGTACTGTGGGTG
P10	TGACAACCGGCTGCGCATTCCTCTGACAATTTCCAAGATGCTGGCGCTCTTTATAAGCAAAAGCGCCCGGTT<br>TTTCAATCCCGTCGATCAAGAAACCGTTACCACGTCCTAAATGGCGCTTTTGCGCCGCCCAACCTGTAAGAG<br>TTACCAGTTACCGCCCCCTCGTGCCGCGCGATGTAATGCATGCATACAAAAGCACAAATCCGAGGACACC<br>ATG
P14	TGATTTTCCGCACGCCGGCGCCGGCCGCGGCCGTTTCATACGGCCGCGCCAGTTTTTTCCATTGCGACCCGC<br>AATTCATCCGATCGTTGATTTTGTCATTAATAATCCCGGAAACGATTTCATAATAAATCCGGAAAAATCGATT<br>ACATAAGTGCCTGCTCCATTTTCAATGATCGCCGGTTTTCTAATAAACGGAATAGTGAATTGGTAAATGTTGA<br>GCAAGGTCACGATGCGCTATGCTGTAAAAAGGTAAAAAGAAAATGCGGGACAGAGTGCCGCGGTATTTTTT<br>TCTCTGCGATGTCGGCGCATCGCAGGCTTGGGCGGCCGCTCGAAATGAAATAAGGGTTTACACCTAGCATTA<br>ATGCCATTTTATTGACAATTAATCGATCTAGAATGACATCCTCCAACTTCCCGTACTCAAATGAGTGGCGGGT<br>TGTCAATAAGTAAGCCGGCTTTTGATCTGGCTTCTATTTTTCTTTACGTCGTCTCTCGCATTGCCAACAGGAG<br>CGTGCCCGATG
P15	CATGCGGACGGTTCCAAAAACGGCGCCCCCAGGCGGGGCGCCCGAAAAAGTTTCAAGGTGAAAAAGCTG<br>CGCGCGACGAACGACGATGGCGCAGATGACGTAGCAGACTAATCAGGGACAATTCAGCCGGTGTGCCCCG<br>CGCGGATTCGCTCCGTCACGGTGCGCAGCGCCCGTTATCTGTTTATAATCAAAGCAATTTTAAAGGATTCGA<br>AGGTGGTTGTATG
P17	TAACCCGCAGCAGCGAACCCGTCACATGATGGAGCAGCCGGCGGCCTCTCGGCCCCGGCCCAAGGTTTAG<br>GAGCAAGAACGCCTGATGCAAAGCATGCCCGGTGCCTTAGCGGCCCAACGACACGACAGGCACTTTGCGA<br>ACCCTTCAGTGGGATGGGCAAACTTCCCTCCGGATAAAGCGGACGGCCGTGTGGCGGTCCGCTTGATCAAG<br>CGATTAGGGGTAGCGGCGCGAGGTGCGCCGGCTACCGACTTGATTTGGCGCGCGAGGCCATGTGGTCACGC<br>GCGCCATACCGTATTCATTAGCGTAAGCGCGCGGTATCCGCGTACGCCGATGAATAGCCCGCTTCGAAGCGC<br>ACGTCCTGAACAGCGTCCGCGGGAAGCGGGTTTTGACGAAGGGTGTAGTTTGAACTGACTCTCATCTGAAC<br>CTGAAGGAGAACAACATG
P18	TAACGACTTGATCGAACCCGTGGAACAGTAGCGACCGCCCCCGAATACGAAACGACGCGGGAAGGCACC<br>GCTCGCCCATCGTTTCACGGCAACCCGGCGATGGGGGCACGTCGCGCACGGCTCCGCCCACCAAGAAACA<br>AATTCCACCATTCCACATTAAAAGAAAACGTTATTCCATATCAAGCTGGATCACCGACCTATTTTGGATCCAT<br>TTTCCCTCCTGTCCCCCGTATCATTTCTCCCATTCAGAATCAGCAAGAAACGGACATG
P19	TGACCCGCCCGCAAGCGCCGTCGCCGCCCGATTTGCCGCCCTTGTTCCTCGCACGCCGACCCGCGCGGGGC<br>GCGTCGGCGCGCGGCCGCCGATCCGATCGGTCCGTTGGTGCGCAGCGACACCGCCACCCCGATTTGACGCT<br>CTTTCACGCACGAACGAAAAGGGGTTGTAATCCGCTGTGATATAACTGCCGACGTGATCCGCTCCTTGAGC<br>GAGACTCACTCCGAACGTCATCCGATGGAGAATAACGATGCAAAAACGGAATCTTGTG

The underlined letters are the initiation and termination codons.

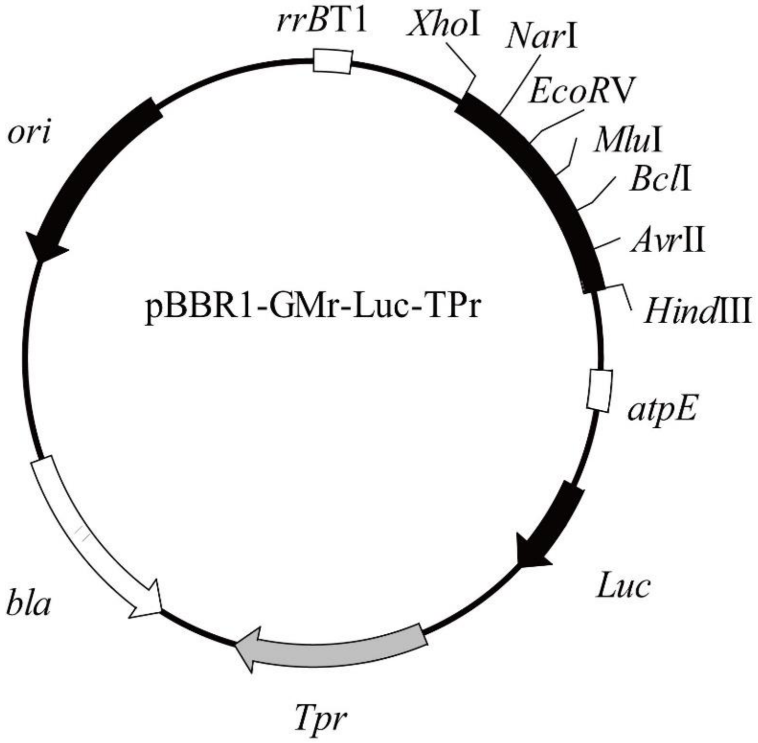

Figure S1. Trap vector with two reporter genes. The vector contains the Tp-resistant dihydrofolate reductase gene (*TP*r) and the luciferase gene set (*Luc*). The most relevant restriction sites that can be introduced into the promoter region are provided. *ori*, origin of replication; *bla*, gene encoding the ampicillin resistance (AmpR) protein; *atpE*, translation initiation region; *rrnBT1*, transcriptional terminator.

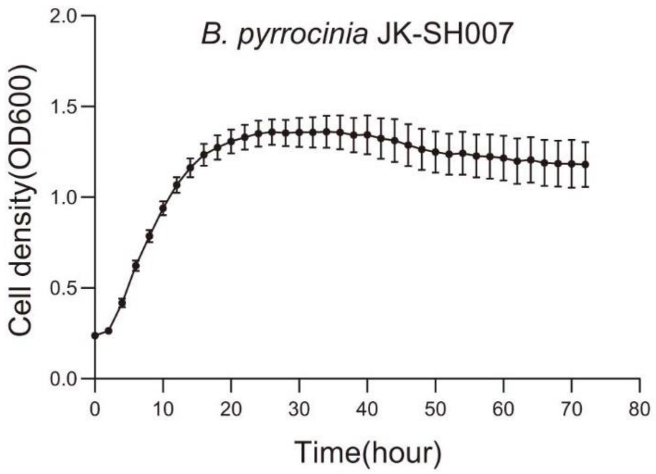

Figure S2. Growth curves of *B. pyrrocinia* JK-SH007. *B. pyrrocinia* JK-SH007 were cultured on LB medium. The standard deviations of the 10 independent replicates are indicated by the error bars.

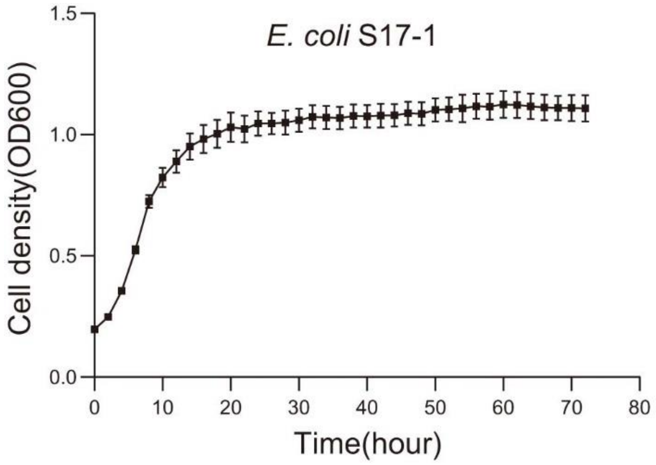

Figure S3. Growth curves of *E. coli* S17-1. *E. coli* S17-1were cultured on LB medium. The standard deviations of the 10 independent replicates are indicated by the error bars.

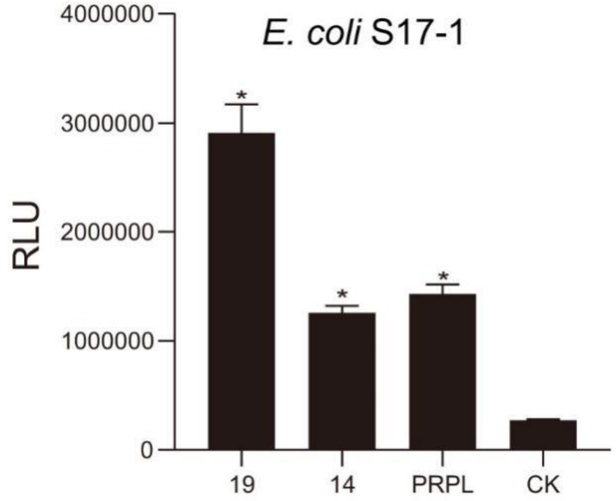

Figure S4. Assay of constitutive promoters from *E. coli* S17-1 using firefly luciferase. PRPL was used as the control variable. CK was a luciferase-specific positive control. The standard deviations from three separate replicates are indicated by the error bars. The data represent means ± SD ($n = 4$). * $p < 0.05$.

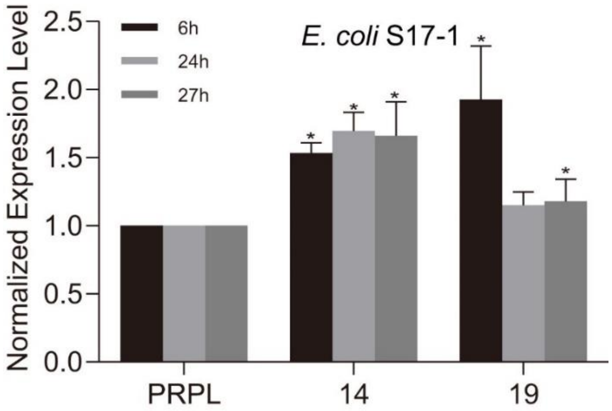

Figure S5. Promoter characterization via qPCR analysis in *E. coli* S17-1. In three different periods, the transcription of the firefly luciferase gene under strong promoters in JK-SH007 was measured. The y-axis depicts the expression value of PRPL, which was set to 1. The standard deviations from three separate replicates are indicated by the error bars. The data represent means ± SD ($n = 4$). * $p < 0.05$.

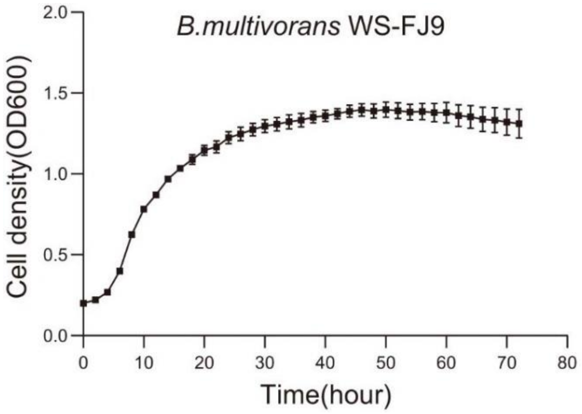

Figure S6. Growth curves of *B. multivorans* WS-FJ9. *B. multivorans* WS-FJ9 were cultured on LB medium. The standard deviations of the 10 independent replicates are indicated by the error bars.

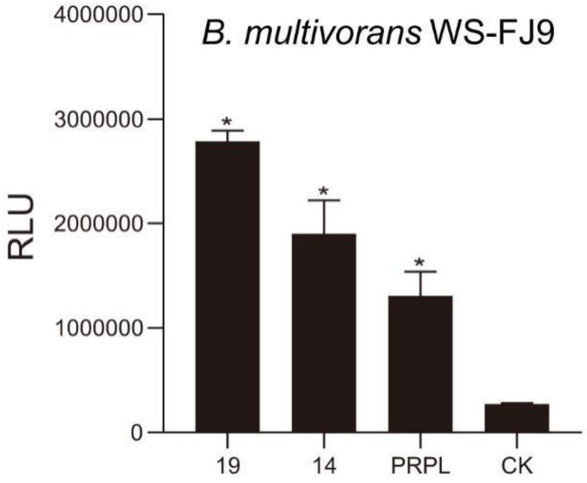

Figure S7. Assay of constitutive promoters from *B. multivorans* WS-FJ9 using firefly luciferase. PRPL was used as the control variable. CK was a luciferase-specific positive control. The standard deviations from three separate replicates are indicated by the error bars. The data represent means ± SD (n = 4). * $p < 0.05$.

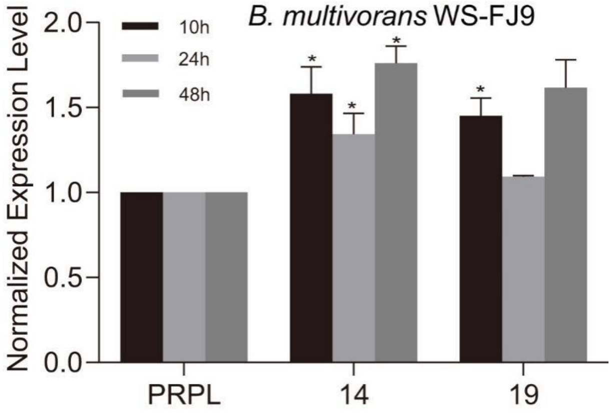

Figure S8. Promoter characterization via qPCR analysis in *B. multivorans* WS-FJ9. In three different periods, the transcription of the firefly luciferase gene under strong promoters in JK-SH007 was measured. The y-axis depicts the expression value of PRPL, which was set to 1. The standard deviations from three separate replicates are indicated by the error bars. The data represent means ± SD (n = 4). * $p < 0.05$.

References

1. Kovach, M.E.; Elzer, P.H.; Hill, D.S.; Robertson, G.T.; Farris, M.A.; Roop, R.M.; Peterson, K.M. Four new derivatives of the broad-host-range cloning vector pBBR1MCS, carrying different antibiotic-resistance cassettes. *Gene* **1995**, *166*, 175–176, https://doi.org/10.1016/0378-1119(95)00584-1.
2. Tomlin, K.L.; Clark, S.R.; Ceri, H. Green and red fluorescent protein vectors for use in biofilm studies of the intrinsically resistant Burkholderia cepacia complex. *J. Microbiol. Methods* **2004**, *57*, 95–106, https://doi.org/10.1016/j.mimet.2003.12.007.
3. He, L.-M.; Ye, J.-R.; Ren, J.-H.; Huang, L.; Wu, X.-Q. Enhancement of antifungal activity of Burkholderia pyrrocinia JK-SH007 genetically modified with Bacillus subtilis Chi113 gene. *For. Pathol.* **2016**, *46*, 632–642, https://doi.org/10.1111/efp.12283.
